# Dementia‐Related Stigma Among Primary School Teachers in Brazil: A Path Toward Public Policy Implementation on Education for Aging

**DOI:** 10.1002/alz70860_105471

**Published:** 2025-12-23

**Authors:** Karen Leticia Pulgatti, Leticia Fernanda Palma, Giulianna Bueno Denari, Lucas Nogueira de Carvalho Pelegrini, Lea da Silva Veras, Márcia Regina Cominetti

**Affiliations:** ^1^ Federal University of São Carlos, São Carlos, São Paulo, Brazil; ^2^ Federal University of São Carlos, São Carlos, SP, Brazil; ^3^ UFSCar, São Carlos, São Paulo, Brazil

## Abstract

**Background:**

Nearly two‐thirds of healthcare professionals still perceive dementia as a normal part of aging, as reported by Alzheimer's Disease International (ADI) in 2019 and 2024. Considering that education plays a critical role in shaping knowledge and attitudes and preparing individuals to engage with societal challenges, including the realities of aging, we investigated dementia‐related stigma among primary school teachers in Brazil. This study aimed to inform targeted educational strategies to reduce stigma, foster understanding, and integrate aging‐related themes into school curricula. The ultimate goal is to provide evidence‐based recommendations for public policies that promote education on aging, address social inequities, and equip younger generations to interact with an aging population more inclusively and knowledgeably.

**Method:**

The study adhered to all ethical guidelines and was registered under the protocol number CAAE: 58365722.2.0000.5504A. We conducted a cross‐sectional study utilizing Google Forms to distribute a structured questionnaire adapted from the ADI instrument, Knowledge and Attitudes Toward Dementia. The survey targeted 63 primary school teachers across a city in Brazil.

**Result:**

Most participants (82.5%) were female, with an average age of 42.3. Their education ranged from Teaching Certification to Postdoctoral degrees, and 47.6% had over 10 years of experience. Additionally, 82.5% had not received training on aging. The ADI Stigma questionnaire revealed that 47.6% perceived individuals with dementia as impulsive and unpredictable, 36.5% were neutral about their potential danger, and 42.8% supported forced medical treatment.

**Conclusion:**

The study highlights significant knowledge gaps and stigma among Brazilian primary school teachers regarding dementia. Misconceptions, such as perceiving individuals with dementia as unpredictable or requiring forced treatment, emphasize the need for targeted educational programs. Integrating aging‐related themes into teacher training and curricula is crucial to reducing stigma, promoting inclusivity, and informing public policies that prepare future generations to engage with an aging population.

